# Designing a Monitoring Program to Estimate Estuarine Survival of Anadromous Salmon Smolts: Simulating the Effect of Sample Design on Inference

**DOI:** 10.1371/journal.pone.0132912

**Published:** 2015-07-21

**Authors:** Jeremy D. Romer, Alix I. Gitelman, Shaun Clements, Carl B. Schreck

**Affiliations:** 1 Oregon Department of Fish and Wildlife, Fish Research, Corvallis, Oregon, United States of America; 2 Oregon State University Department of Statistics, Corvallis, Oregon, United States of America; 3 United States Geological Survey, Oregon Cooperative Fish and Wildlife Research Unit, Oregon State University, Corvallis, Oregon, United States of America; University of Aveiro, PORTUGAL

## Abstract

A number of researchers have attempted to estimate salmonid smolt survival during outmigration through an estuary. However, it is currently unclear how the design of such studies influences the accuracy and precision of survival estimates. In this simulation study we consider four patterns of smolt survival probability in the estuary, and test the performance of several different sampling strategies for estimating estuarine survival assuming perfect detection. The four survival probability patterns each incorporate a systematic component (constant, linearly increasing, increasing and then decreasing, and two pulses) and a random component to reflect daily fluctuations in survival probability. Generally, spreading sampling effort (tagging) across the season resulted in more accurate estimates of survival. All sampling designs in this simulation tended to under-estimate the variation in the survival estimates because seasonal and daily variation in survival probability are not incorporated in the estimation procedure. This under-estimation results in poorer performance of estimates from larger samples. Thus, tagging more fish may not result in better estimates of survival if important components of variation are not accounted for. The results of our simulation incorporate survival probabilities and run distribution data from previous studies to help illustrate the tradeoffs among sampling strategies in terms of the number of tags needed and distribution of tagging effort. This information will assist researchers in developing improved monitoring programs and encourage discussion regarding issues that should be addressed prior to implementation of any telemetry-based monitoring plan. We believe implementation of an effective estuary survival monitoring program will strengthen the robustness of life cycle models used in recovery plans by providing missing data on where and how much mortality occurs in the riverine and estuarine portions of smolt migration. These data could result in better informed management decisions and assist in guidance for more effective estuarine restoration projects.

## Introduction

A number of salmonid populations are at risk of extinction throughout much of their range [1–5]. Declines have primarily been attributed to habitat loss and the effects of hydropower, hatcheries, and harvest [6–8]. However, the lack of baseline data often hinders attempts to isolate the factors causing population declines [7]. Long term monitoring of population health is required for the classification of existing degrees of peril, identification of problem areas, and evaluation of recovery measures. To address these needs, a number of U.S. states have instituted long term monitoring programs to assess trends in smolt to adult survival [9–12]. However, none of these long-term programs currently incorporate monitoring to estimate smolt survival through the estuary into the near shore marine zone; an area in which substantial loss has been documented [5], [13–22].

Estimating the survival of juvenile salmon as they pass through the estuary has been hindered by an inability to count fish as they enter and exit these large, dynamic environments. To address this issue there has been increased use of biotelemetry to estimate the survival of outmigrating salmonids in the past two decades [23], though typically the studies are only 1–2 years in duration [5], [15–17], [19], [21]. These studies have identified a general pattern of high smolt mortality in the estuary, and in some cases, results have been used to guide management actions to limit the effects of predators on threatened salmon stocks (e.g., [24]). However, there has been little discussion about how the design of telemetry studies could affect the interpretation of survival data, and subsequent inference to the salmonid population in question. This information is particularly important when evaluating the utility of biotelemetry as a tool for long term monitoring of smolt survival.

To address this issue, we developed simulations for several conceptual sampling designs in which acoustic-tagged fish were released in one or more groups over the course of a simulated outmigration. Sampling design refers to the method by which smolts were selected, or sampled, from the larger migrant population prior to tagging. The purpose of simulating different sampling designs was to test whether the sampling design (number of tags, time of tagging) influenced the bias in survival estimates given changes in daily smolt survival and run timing distributions observed in nature. The simulations were not intended to answer specific research questions (behavior, habitat utilization, migration rates, temporal survival), or to evaluate all permutations of study design and environmental variability. Instead, we wanted to highlight factors that should be taken into consideration prior to using biotelemetry as a tool to monitor annual outmigration survival over multiple years. Our simulations included scenarios in which the outmigrant run distribution and daily survival varied. We compared the estimates of survival for the outmigrant population among these different models to determine the effect of sample size and timing of tagging on the accuracy and precision of survival estimates. The simulation parameters were based on observations from empirical, field based studies of steelhead (*Oncorhynchus mykiss*) smolt survival using acoustic telemetry in Oregon, USA. Our results can be used to inform both researchers and managers about the potential effects of sampling design when using acoustic telemetry to measure estuarine mortality in outmigrating salmonids.

## Methods

### Simulations

We performed a simulation study to evaluate how different sampling designs and sample sizes might affect estimates for survival probability. In the simulation, we consider two run distribution patterns and four survival probability patterns; these are described next.

### Run Distributions

**Fig 1:**
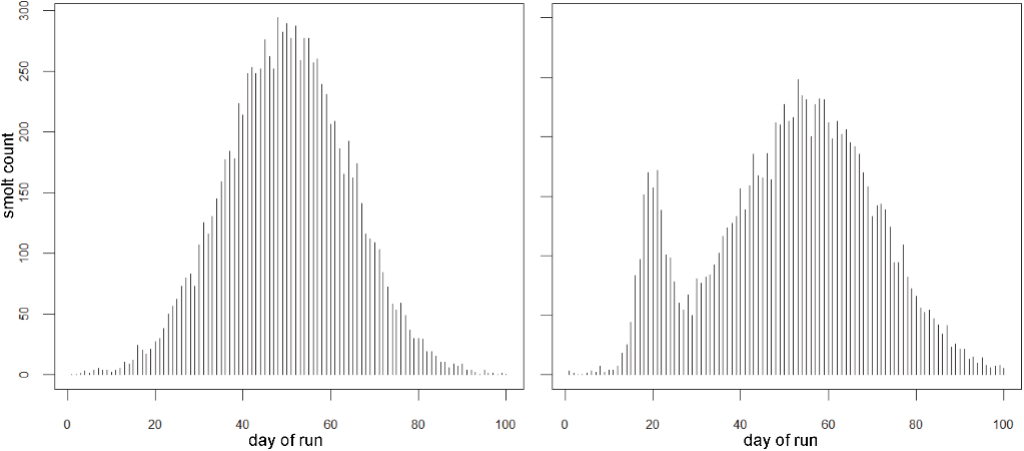
Two simulated run distribution patterns modeled using a population size of 10,000 smolts and run duration of 100 days. Simulations are based on patterns observed in nine years of smolt trapping data (1999–2007) from seven ODFW Life Cycle Monitoring sites on the Oregon Coast.

We obtained outmigrant smolt trapping data from seven Life Cycle Monitoring sites on the Oregon coast during the period 1999–2007 from the Oregon Department of Fish and Wildlife (ODFW) [25]. We observed two common run distribution patterns in these data, one that was roughly unimodal and symmetric (accounting for almost 90% of observed runs) and one that was bimodal, with a smaller pulse of smolts followed by a larger pulse S1 Fig. In our simulated versions of these distributions, we assumed a duration of 100 d and an outmigrating population of ~10,000 smolts (Fig 1). Specifically, for the unimodal distribution we generated Poisson rate parameters proportional to the height of a normal probability density function (pdf) for each of the 100 days of the run. Then, using these rate parameters, we simulated daily run counts from Poisson distributions. For the bimodal distribution, the rate parameters were generated to be proportional to the height of two normal pdfs S1 Text.

### Survival Probability Patterns

**Fig 2:**
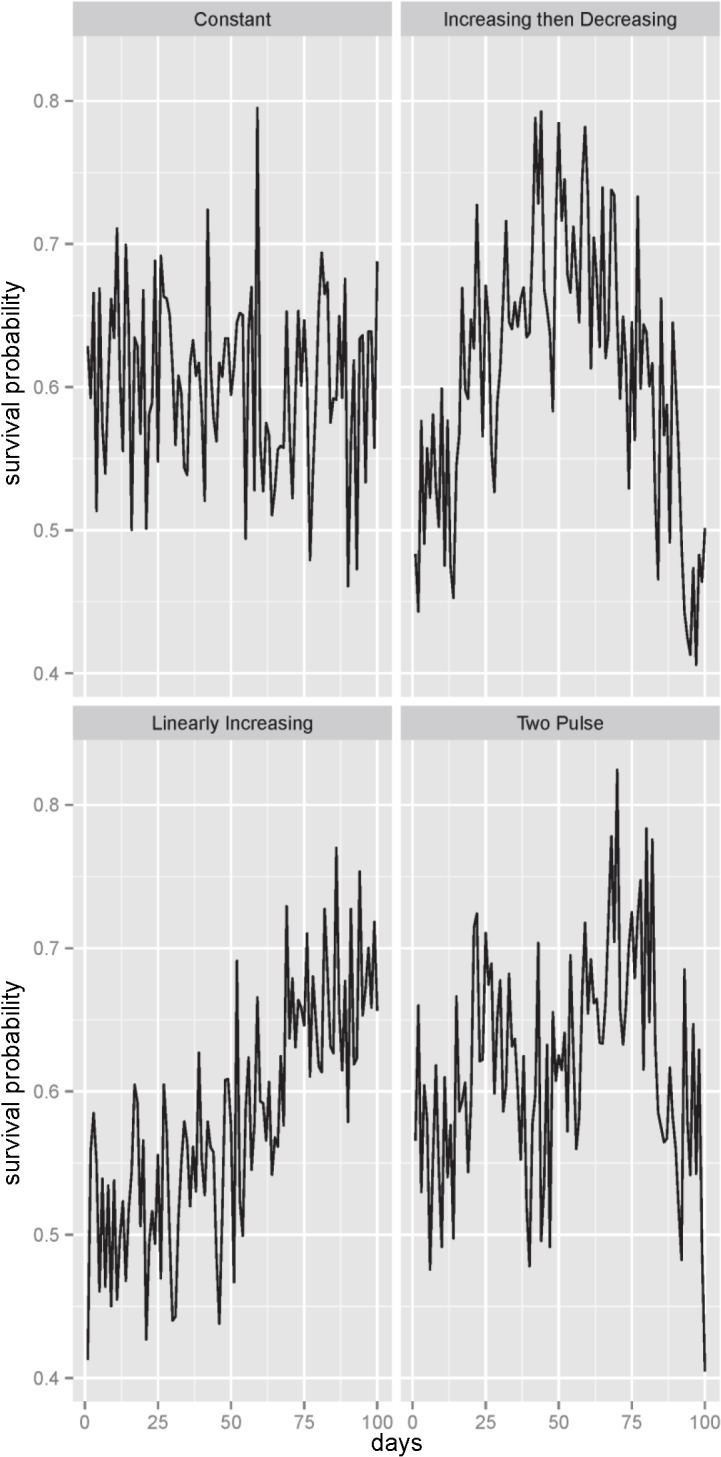
Survival probability patterns, each with added day-to-day fluctuations (*σ* = 0.06). All survival probabilities are generally between 0.4 and 0.8, and the seasonal average survival probability is about 0.6 for all patterns.

We used data previously collected using biotelemetry in the Nehalem and Alsea rivers[13], [15], [20] to suggest general patterns for survival probabilities throughout runs (constant, linearly increasing, and symmetrically increasing and then decreasing), as well as a baseline range for the survival probabilities (seasonally, these probabilities ranged from 0.4 to 0.8). Additionally, we included a two-pulse pattern where the survival probability increases then decreases twice throughout the duration of the run. Although this two-pulse pattern was not observed, it remains conceptually feasible. Prior data [14], [17] suggest that survival while migrating through an estuary is highly variable, even between consecutive days. To account for this, we incorporated random noise for each survival probability pattern, while keeping the survival probabilities generally in the 0.4 to 0.8 range. The noise component was zero-centered normal noise with standard deviation of 0.06. The four patterns of survival probability are shown in Fig 2.

### Sampling Designs and Sample Sizes

We compared survival estimates from four sampling designs, each with four different sample sizes, *n* = 12, 20, 100, and 1000 (sample size = total number of acoustic tags used in a study). We chose these values because 1) in many coastal systems it is not possible to capture large numbers of wild outmigrating smolts on a single day and 2) the cost of tags may become prohibitive at higher *n* values. For the sake of comparison, we ran simulations for n = 1000 even though this is an unrealistic number of tagged smolts for the systems we considered. In one design, all *n* fish are captured and tagged on a single day that is randomly chosen from the peak of the run (we call this PEAK), where the peak is defined as between days 40 and 60 of the outmigration. Two sampling designs (SYST1 and STST2) use systematic sampling: a day is chosen at random from the start (defined to be between days 20 and 40 after the first smolt is captured) of the run, and then three subsequent days at equally spaced intervals are also chosen. On each of the four days, *n*/4 fish are sampled and tagged. In SYST1, the four tagging days are spaced 7 d apart, whereas in SYST2, the spacing is 14 d. We chose these intervals as researchers have commonly used 1–2 week intervals between tagging events spread across the outmigration. In the final sampling design, RAND, four days were chosen at random from the entire length of the run, and *n*/4 fish were “tagged” on each of the four days. Our expectations were that the RAND sampling design would produce the most accurate estimates and that the SYST designs would outperform the PEAK.

### Simulation Steps

We compared the four sampling designs and three sample sizes by generating 1,000 survival probability estimates for each combination of survival probability pattern, sampling design, and sample size, for a total of 4 × 4 × 4 = 64 simulation runs. Specific details for each sampling design are described next.

For the PEAK sampling design we:

1. Randomly selected one day between days 40 and 60.
2. Obtained the survival probability corresponding to the selected day from each of the four patterns of survival probabilities in Fig 2.
3. Used that survival probability to randomly generate a binomial count for each of the sample sizes *n* = 12, 20, 100, and 1000.
4. Calculated survival probability estimates as the counts in step 3 divided by the corresponding sample size, *n*.
5. Repeated 1000 times.

For the SYST sampling designs:

1. We randomly selected a start day, D, between days 20 and 40.
2. For each survival probability pattern in Fig 2 we:
  - Obtained survival probabilities corresponding to days D, D+7, D+14, and D+21
  - Obtained survival probabilities corresponding to days D, D+14, D+28, and D+42
3. For each sampling day, we randomly generated a binomial count with sample sizes equal to *n*/4 for *n* = 12, 20, 100, 1000.
4. We calculated survival probability estimates as the sums of the four binomial counts divided by the corresponding sample size, *n* = 12, 20, 100, 1000.
5. The procedure was repeated 1000 times.

For the RAND sampling design:

1. We randomly selected 4 days between 1 and 100
2. For each survival probability pattern in Fig 2, we obtained survival probabilities corresponding to the selected days
3. For each selected day, we randomly generated a binomial count with sample size equal to *n*/4.
4. We calculated survival probability estimates as the sum of the four binomial counts divided by the corresponding sample size, *n* = 12, 20, 100, 1000.
5. The procedure was repeated 1000 times.

We evaluated these results in terms of bias and variation, and by examining confidence interval coverage. For each survival probability estimate, we also constructed 95% confidence intervals using the Wilson interval as recommended by Brown et al. 2001 [26]. By examining the confidence interval coverage (i.e., frequency of overlap between the seasonal survival probability and the simulated confidence intervals), we evaluated the performance of each sampling design at estimating survival probability for the entire season. By “valid”, we mean that a 95% confidence interval constructed from the simulation data covers the true survival probability (known to us in the simulations) in 95% of simulations.

### Expected Number of Surviving Smolts

We also evaluated the influence of sampling design on the accuracy of estimating the number of smolts surviving to the ocean. To do this, we incorporated the outmigrant numbers derived from the run distributions in Fig 1. We estimated the number of smolts sucessfully entering the ocean by multiplying the total run size by the estimated survival probability for the entire season. Using both the total run size and the shape of the run distribution (as in Fig 1), we hypothesized that we could improve upon the estimate of expected count of surviving smolts by using the sampling designs which provided estimates of survival probability from four different days during the run (namely, SYST1, SYST2 and RAND). That is, rather than taking a single survival probability to represent the survival probability for the entire season, if we used the four separate estimates to integrate over the differently shaped run distributions it should improve estimates of expected survival counts.

## Results

The bias estimates from 1000 simulations of the four sampling designs and the four survival probability patterns for the sample size *n* = 20 are given in Table 1. Results for *n* = 12 and *n* = 100 were quite similar. The bias was highest under the increasing then decreasing survival pattern for all sampling designs and lowest under the RAND sampling design for all survival patterns. In general, our expectations were validated—the RAND design outperformed the others in terms of minimizing bias, and the two SYST designs generally outperformed the PEAK design. In terms of the survival probability patterns, it was also not surprising that the lowest bias values were associated with the constant survival pattern. The two pulse survival pattern also yielded relatively low bias values across all sampling designs.

**Table 1:** Mean bias due to survival probability pattern and sampling design from 1000 simulations of n = 20 observations at each setting. Each entry is the mean difference between estimated survival probability and the true season-averaged survival probability used for the 1000 simulations at each setting. Bias results are comparable for the other sample sizes. For a sample size of n = 20, a typical standard error for the proportion estimate is on the order of 0.10; for a sample size of 100, it is on the order of 0.05.

	Sampling Design			
Survival Probability Pattern	Peak	Syst 1	Syst 2	Rand
Constant	0.019	0.010	0.001	0.001
Linearly Increasing	-0.024	-0.031	-0.004	-0.002
Increase/Decrease	0.090	0.063	0.052	0.005
Two Pulse	-0.010	-0.004	0.026	0.000

It is important to note that there was substantial variation in the survival probability estimates across simulations. For samples sizes of *n* = 12, 20, 100 and 1000, the standard deviation across simulations was generally 0.15, 0.11, 0.05, and 0.03 respectively. Taken together with our observation that the bias remains stable across different sample sizes, this suggests the rather counter-intuitive result that large sample sizes actually increase the likelihood of obtaining a confidence interval that does not cover the true underlying season-averaged survival probability.

In addition to examining the point estimates, we considered the confidence interval coverage for the different sampling designs. In simulations, the 95% confidence interval coverage was defined as the percentage of times (out of 1000 simulations) that the confidence intervals covered the true parameter value. If the sampling procedure performed as expected the percentage should be approximately 95%. Results are shown in Table 2. There are several important things to notice. First, very few of the sampling designs achieved 95% coverage at any of the sample sizes, with the coverage generally too low (< 95%). This is likely a function of using simple sample proportions as estimates of survival probability. Using this approach, we essentially only estimated the smooth functions underlying the different survival probability patterns in Fig 2. That is, our simple estimates (though no different from what would typically be used assuming 100% detection) substantially under-estimated the day-to-day variation in survival probability, and this was reflected in the poor confidence interval coverages. Second, the coverage results corresponding to the increasing then decreasing survival probability patterns were especially poor relative to the other survival probability patterns. Third, the coverages for the larger sample sizes (n = 100 and n = 1000), were substantially lower than those for the smaller sample sizes. This may seem counterintuitive until one considers that the larger sample sizes result in much narrower confidence intervals. The interval widths for *n* = 12, 20,100 and 1000 were ~ 0.47, 0.38, 0.18, and 0.06, respectively. It seems clear that with the larger sample sizes we substantially underestimated the standard error. Finally, the two systematic sampling designs had the best overall performance in terms of confidence interval coverage, except in the case of the increasing then decreasing survival pattern where the random sampling design resulted in slightly better coverage.

**Table 2:** Coverage percentages for different survival probability-sampling design combinations using the Wilson interval (nominal coverage is 95%).

Survival Probability Pattern		Sampling Design			
	Sample Size	PEAK	SYST1	SYST2	RAND
Constant	*n* = 12	92.7	95.4	96.0	95.8
	*n* = 20	92.8	97.1	95.7	95.7
	*n* = 100	77.3	93.1	90.7	90.7
	*n* = 1000	39.1	74.5	65.4	60.9
Linearly Increasing	*n* = 12	95.3	95.0	96.0	95.0
	*n* = 20	89.2	91.2	91.4	93.6
	*n* = 100	76.6	81.7	90.3	87.0
	*n* = 1000	39.4	38.1	63.0	50.7
Increasing, Decreasing	*n* = 12	89.7	92.8	94.0	92.5
	*n* = 20	83.6	92.7	93.8	95.4
	*n* = 100	47.6	72.9	78.1	84.3
	*n* = 1000	9.0	17.3	18.9	47.2
Two Pulse	*n* = 12	90.6	91.0	93.5	92.7
	*n* = 20	89.1	94.1	93.9	92.2
	*n* = 100	75.7	92.1	90.0	87.4
	*n* = 1000	36.5	62.7	56.0	56.4

Using the two different run-distribution shapes (unimodal, bimodal), we evaluated the different sampling strategies in combination with the four survival probability patterns in terms of how well they provided estimates of expected survival counts. The results for estimating expected counts are fairly similar to those for the survival probability estimates. There were no clear differences in the performance of the sampling designs relative to the shape of the underlying run distributions. For estimating expected counts, the confidence coverage for all sampling designs remained low (<95%), though the coverage for the systematic designs were marginally better than for the random design. The peak sampling design typically had the lowest coverage.

## Discussion

Our simulations illustrate the effect that sampling design can have on estimating outmigrant salmonid smolt survival through an estuary under scenarios that represent commonly observed run distribution and survival patterns. Unfortunately, none of the four sampling design scenarios that we simulated were sufficiently accurate from a statistical perspective. The 95% confidence limits around our simulated estimates encompassed the true mean less than 95% of the time across most the sampling/survival probability pattern scenarios we evaluated. It is possible that these methods could be further refined with additional modeling to include a component for day to day variation, but the larger issue is that the underlying pattern of survival probability has a significant effect on the bias, and is typically unknown in a field study.

Several studies have documented substantial mortality (14–77%) during outmigration through the estuary [5], [13], [15], [16], [18–22]. If these reported survival estimates are accurate, current models of life cycle survival have likely over-estimated the level of ocean mortality (over which managers have little control). Given this, the implementation of long term monitoring of estuarine smolt survival has several potential benefits. These include improved models of life history survival (by assigning a potentially large proportion of mortality to where it actually occurs), improved data to guide plans for where estuarine restoration may be needed (by identifying estuaries with consistently high mortality rates), the ability to measure the success of such restoration efforts (by analyzing trends in survival prior and subsequent to restoration efforts), and potentially improved forecasting of adult returns (by incorporating estuarine smolt survival in current recruitment models).

Our results were encouraging for implementation of a large scale, long term monitoring program in multiple representative basins within each management area to detect trends in survival among years. The relatively small number of tags (12–20) needed to achieve adequate (based on currently accepted 95% confidence intervals within ± 30% of the mean in the Oregon Plan for Salmon and Watersheds) [27–29] survival estimates results in minimal capital investment required for such monitoring. However, we caution that these numbers are only a starting point. The actual number to be tagged should also account for the anticipated loss of tagged fish prior to reaching the estuary and the anticipated detection probability of arrays. In regard to the design for *when* the smolts should be tagged, our simulations suggest that the sampling effort is most effective when it is spread out over some period of the run—generally the systematic and random sampling designs resulted in the most accurate estimates. Our results also indicate that the pattern of the survival probabilities throughout the course of the run can be quite important for the performance of the survival probability estimates. Unfortunately, these patterns are not typically known, but this could be a new area for investigation.

Smolts tagged upstream of the estuary on the same day distribute themselves throughout the lower river as they migrate downstream. Several factors influence the distribution of tagged fish in the lower river (e.g., stream discharge at the time of release and distance between tagging site and the estuary). Therefore, groups of smolts tagged on the same day enter and migrate through the estuary over a period of time. The survival estimates from these fish provide an estimate that will be averaged over the period when they are migrating through the estuary. This temporal averaging is not incorporated in our simulation.

The primary objective of this manuscript was to illustrate the influence of sampling design (number of tags, time of tagging) on bias associated with estimates of outmigration survival. As such, the detection efficiency of acoustic arrays is a confounding factor that we did not consider. Detection efficiency is known to vary substantially over short periods with changes in environmental noise, resulting in an increase in the size of the confidence intervals associated with survival estimates. This variation in detection efficiency can be mitigated to a large extent by ensuring adequate coverage of acoustic arrays (spacing and number of receivers). However, we acknowledge that achieving perfect detection in a field situation is nearly impossible, and that assuming perfect detection for an actual survival study would result in erroneous survival estimates.

There are also considerations that need to be addressed when using a RAND-type sampling design. For instance, there is a possibility of generating or drawing “unacceptable” random samples. If each of the four random sampling dates were very close together, or if they incorporated tagging dates at the very beginning or end of the run then we would consider this to be an unacceptable option. Based on our prior observations, we also suggest that *O*. *mykiss* should not be tagged at the very beginning, or the end of the migration to estimate survival for a steelhead smolt population. Juveniles captured at either extreme (early, late) may merely be moving short distances to feed or rear, and not migrating [13]. The period of acceptable tagging dates becomes especially important when fish availability is limited (due to small run sizes or poor trapping efficiency), or trapping effort is delayed during previously scheduled tagging (e.g., high water or severe weather). To help avoid some of these issues, for new study sites where there may be no background data on the number of smolts available we recommend a pilot year of smolt trapping to collect useful data about migration timing, duration of outmigration, smolt abundance, and the size of fish available for tagging.

Scientific method begins with a question. Sampling design and analyses are then predicated on the most appropriate methods to answer the aforementioned question. The focus of our study was to estimate the survival probability for one cohort of smolts migrating through the estuary, and the results from the simulations reported here are specific to this question. A RAND sampling design utilizing a relatively small number of tags would *not* be appropriate, for instance, to detect the effect of a given action (i.e., evaluating efficacy of restoration projects) or detect temporal changes in survival probabilities within a season–which would require increased sampling effort and a study design tailored specifically to answer these research questions.

Prior to this simulation exercise we hypothesized that, regardless of sampling protocol, increasing the number of tags would bring survival estimates closer to the “true survival” realized by a cohort of outmigrating smolts. This was not the case. Increasing the numbers of acoustic tagged smolts increased the *precision* of our estimates, but not the *accuracy*. The simulations illustrate that without a thorough understanding of the underlying variation in survival probabilities, increased precision resulting from tagging more fish will tend to draw the survival estimate further from the actual survival value.

## Supporting Information

S1 Fig Histograms with the number of steelhead smolts captured each year summarized by week (left, colored histograms; 1999–2007) and normalized run distributions summarized by the percent of migrants captured each week throughout the run (right, greyscale histograms). Numbers were not corrected for trap efficiencies and represent raw catch numbers summarized by week. Dotted grey lines symbolize the bimodal distribution with a small pulse of smolts followed by the larger, main peak of the migration. Solid black lines symbolize primarily unimodal run distribution patters. Scales on the axes (x and y) have not been standardized among basins, as number of smolts captured and period of migration are highly variable among basins.
(DOCX)

S1 Text Statistical program R code for running simulations. (DOCX)
