# Supplementary material for: Designing a Monitoring Program to Estimate Estuarine Survival of Anadromous Salmon Smolts: Simulating the Effect of Sample Design on Inference
Source: PLoS One. 2015 Jul 21;10(7):e0132912. doi: 10.1371/journal.pone.0132912 (PMC4510331; doi:10.1371/journal.pone.0132912)
Supplement: S1 Fig — Numbers were not corrected for trap efficiencies and represent raw catch numbers summarized by week. Dotted grey lines symbolize the bimodal distribution with a small pulse of smolts followed by the larger, main peak of the migration. Solid black lines symbolize primarily unimodal run distribution patters. Scales on the axes (x and y) have not been standardized among basins, as number of smolts captured and period of migration are highly variable among basins. (DOCX) [file pone.0132912.s001.docx]

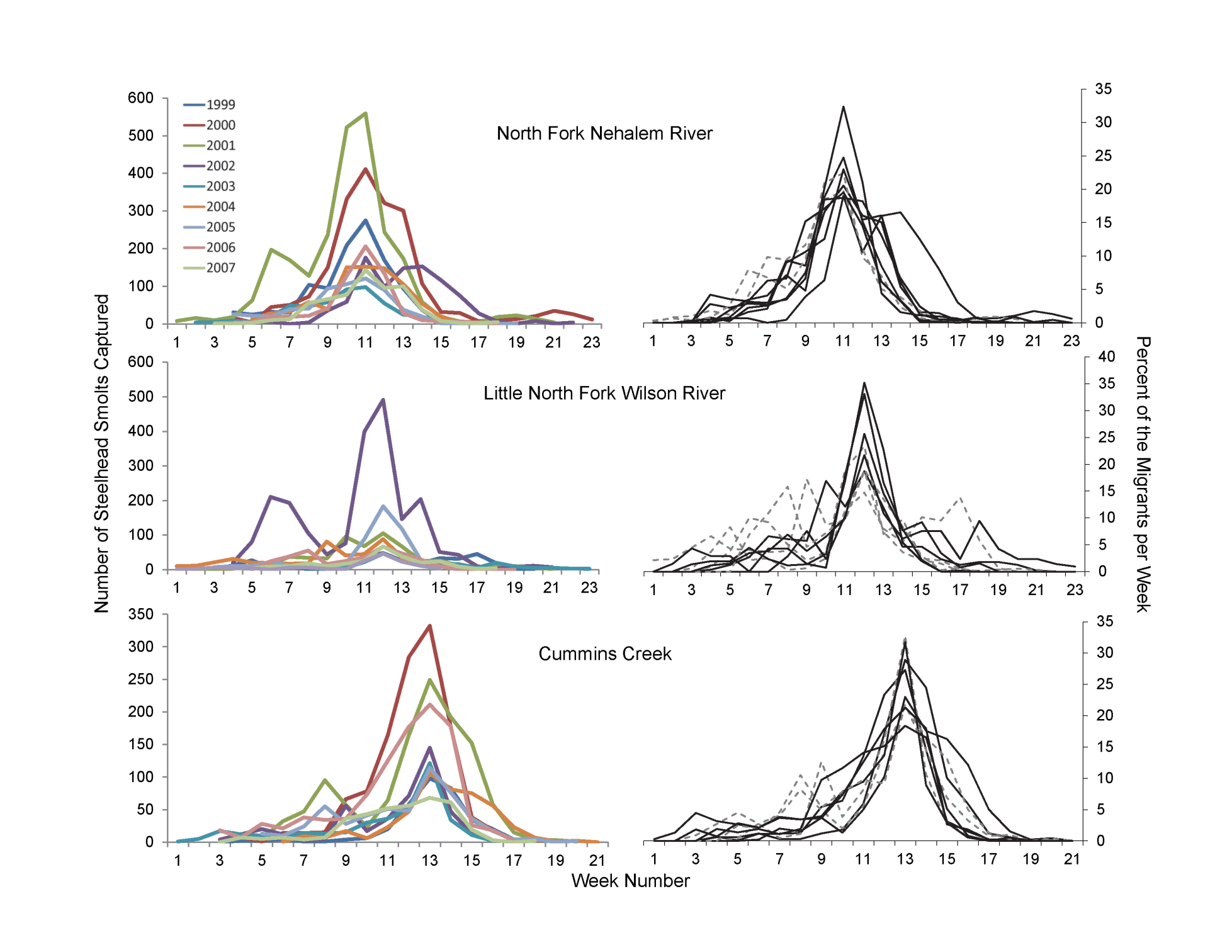

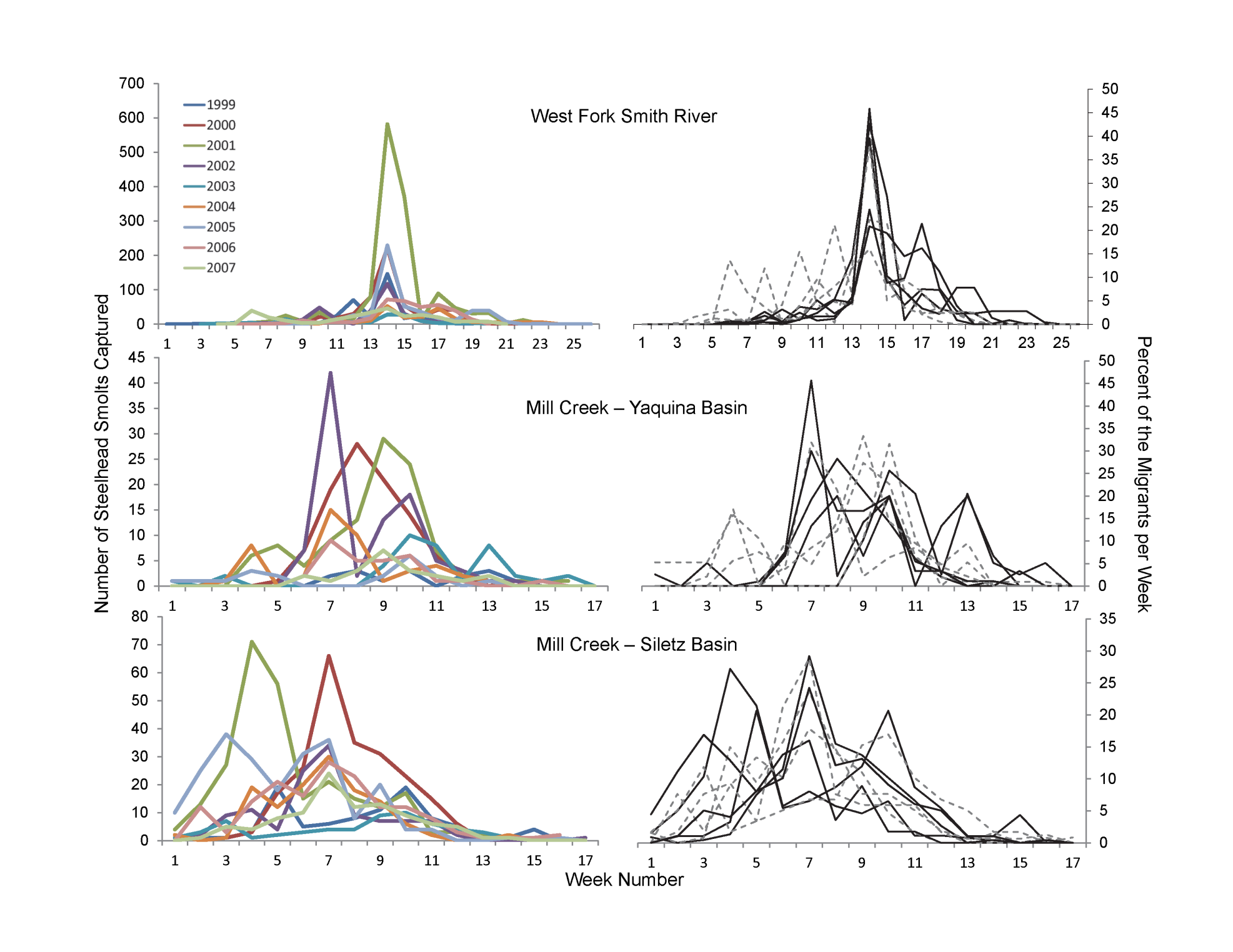

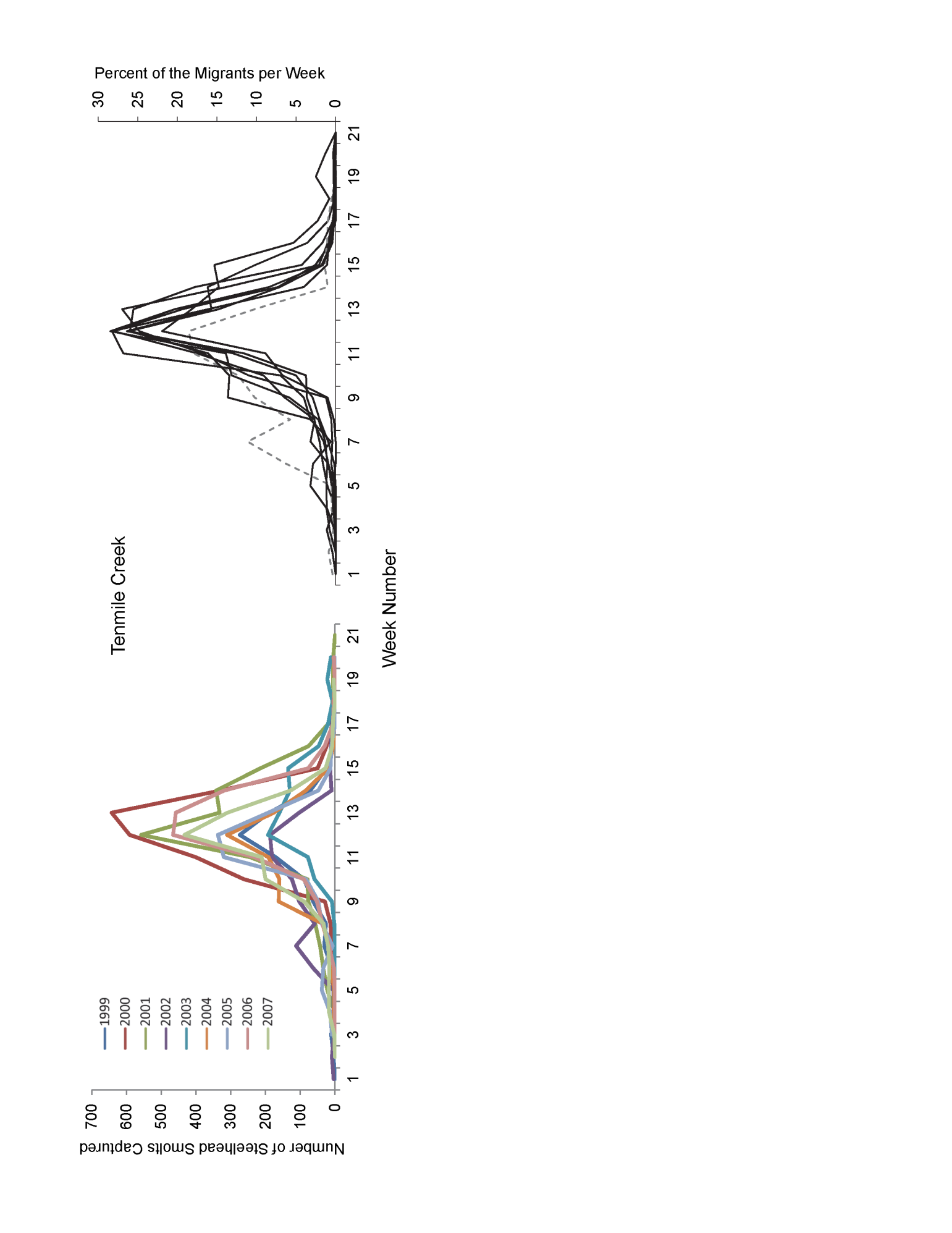


**Figure S1. Histograms with the number of steelhead smolts captured each year summarized by week (left, colored histograms; 1999 – 2007) and normalized run distributions summarized by the percent of migrants captured each week throughout the run (right, greyscale histograms).** Numbers were not corrected for trap efficiencies and represent raw catch numbers summarized by week. Dotted grey lines symbolize the bimodal distribution with a small pulse of smolts followed by the larger, main peak of the migration. Solid black lines symbolize primarily unimodal run distribution patters. Scales on the axes (x and y) have not been standardized among basins, as number of smolts captured and period of migration are highly variable among basins.
