# Supplementary material for: Designing a Monitoring Program to Estimate Estuarine Survival of Anadromous Salmon Smolts: Simulating the Effect of Sample Design on Inference
Source: PLoS One. 2015 Jul 21;10(7):e0132912. doi: 10.1371/journal.pone.0132912 (PMC4510331; doi:10.1371/journal.pone.0132912)
Supplement: S1 Text — (DOCX) [file pone.0132912.s002.docx]

#

# Text S1. R script for simulations

#

#

# Assume a 100-day long season.

#

#

# simulate run distributions

#

tt <- 1:100

set.seed(1)

lam <- dnorm(tt,50,14)

rd1 <- rpois(100,lam*10000) # unimodal RD

set.seed(7)

lam <- 0.1*dnorm(tt,20,3)+0.9*dnorm(tt,55,16)

rd2 <- rpois(100,lam*10000) #bimodal RD

#

# constant survival probabilities

#

D <- 1:100

ES <- matrix(0,4,2)

set.seed(16)

D1 <- rnorm(100,0.6,0.06)

sp1 <- mean(D1)

ES[1,1] <- sum(D1*rd1)

ES[1,2] <- sum(D1*rd2)

#

# linearly increasing survival probabilities

#

s <- 0.25/99

yi <- 0.45

set.seed(14)

D2 <- yi + s*D + rnorm(100,0,0.06)

sp2 <- mean(D2)

ES[2,1] <- sum(D2*rd1)

ES[2,2] <- sum(D2*rd2)

#

# increasing then decreasing survival probabilities

#

a = -0.25/2450

b = -101*a

c = 0.45 - a - b

set.seed(13)

D3 <- a*D^2+b*D+c + rnorm(100,0,.06)

sp3 <- mean(D3)

ES[3,1] <- sum(D3*rd1)

ES[3,2] <- sum(D3*rd2)

#

# two pulse survival probabilities

#

set.seed(15)

D4 <- 0.55 + 0.1*exp(-(D-25)^2/80) + 0.20*exp(-(D-70)^2/170) + rnorm(100,0,0.06)

sp4 <- mean(D4)

ES[4,1] <- sum(D4*rd1)

ES[4,2] <- sum(D4*rd2)

truth = c(sp1,sp2,sp3,sp4)

sp.mat = cbind(D1,D2,D3,D4)

ss = c(12,20,100)

########################################################################

#

# PEAK sampling scheme.

#

########################################################################

set.seed(42)

peak.arr <- array(0,c(4,2,3,1000))

peak.EC <- array(0,c(4,2,3,1000))

#

# survival probability estimates:

#

# dimension 1 is the four survival probability patterns (const,lin,quad,2pulse)

# dimension 2 is the two run distributions (uni,bi)

# dimension 3 is the three sample sizes (12,20,100)

# dimension 4 is the 1000 simulations

#

for (i in 1:4) { # loop through SP functions

for (j in 1:2) { # loop through RD

if(j==1) rdcur=rd1 else rdcur=rd2

for (l in 1:1000) { # simulations

d = round(runif(1,40,60)) # select Day

phat = rbinom(3,ss,sp.mat[d,i])/ss

peak.arr[i,j,,l] = phat

peak.EC[i,j,,l] = phat*sum(rdcur)

}

}

}

#########################################################################

#

# SYST sampling scheme.

#

#########################################################################

set.seed(55)

syst.arr1 <- array(0,c(4,2,3,1000))

syst.arr2 <- array(0,c(4,2,3,1000))

syst.EC1 <- array(0,c(4,2,3,1000))

syst.EC2 <- array(0,c(4,2,3,1000))

ss = c(12,20,100)

ns = c(3,5,25)

for (i in 1:4) {

for (j in 1:2) {

if (j==1) rdcur=rd1 else rdcur=rd2

for (l in 1:1000) {

d = round(runif(1,20,40))

ps1 = sp.mat[c(d,d+7,d+14,d+21),i]

ps2 = sp.mat[c(d,d+14,d+28,d+42),i]

for (k in 1:3) {

phat1 = sum(rbinom(4,ns[k],ps1))/ss[k]

phat2 = sum(rbinom(4,ns[k],ps2))/ss[k]

syst.arr1[i,j,k,l] = phat1

syst.EC1[i,j,k,l] = phat1*sum(rdcur)

syst.arr2[i,j,k,l] = phat2

syst.EC2[i,j,k,l] = phat2*sum(rdcur)

}

}

}

}

#######################################################################

#

# Random Sampling Scheme (select 4 days, randomly between 1 and 100)

#

#######################################################################

set.seed(46)

rand.arr <- array(0,c(4,2,3,1000))

rand.EC <- array(0,c(4,2,3,1000))

#

# survival probability estimates:

#

# dimension 1 is the four survival probability patterns (const,lin,quad,2pulse)

# dimension 2 is the two run distributions (uni,bi)

# dimension 3 is the three sample sizes (12,20,100)

# dimension 4 is the 1000 simulations

#

for (i in 1:4) { # loop through SP functions

for (j in 1:2) { # loop through RD

if(j==1) rdcur=rd1 else rdcur=rd2

for (l in 1:1000) { # simulations

d = round(runif(4,1,100))

ps = sp.mat[d,i]

for (k in 1:3) {

phat = sum(rbinom(4,ns[k],ps))/ss[k]

rand.arr[i,j,k,l] = phat

rand.EC[i,j,k,l] = phat*sum(rdcur)

}

}

}

}
